# Speaking up, support, control and work engagement of medical residents. A structural equation modelling analysis

**DOI:** 10.1111/medu.13951

**Published:** 2019-09-30

**Authors:** Judith J Voogt, Toon W Taris, Elizabeth L J van Rensen, Margriet M E Schneider, Mirko Noordegraaf, Marieke F van der Schaaf

**Affiliations:** ^1^ Executive Board University Medical Centre Utrecht Utrecht University Utrecht the Netherlands; ^2^ Utrecht School of Governance Utrecht University Utrecht the Netherlands; ^3^ Department of Psychology Utrecht University Utrecht the Netherlands; ^4^ Centre for Research and Development of Education University Medical Centre Utrecht Utrecht University Utrecht the Netherlands

## Abstract

**Objectives:**

Medical residents can play key roles in improving health care quality by speaking up and giving suggestions for improvements. However, previous research on speaking up by medical residents has shown that speaking up is difficult for residents. This study explored: (i) whether two main aspects of medical residents’ work context (*job control* and *supervisor support*) are associated with speaking up by medical residents, and (ii) whether these associations differ between in‐hospital and out‐of‐hospital settings.

**Methods:**

Speaking up was operationalised and measured as voice behaviour. Structural equation modelling using a cross‐sectional survey design was used to identify and test factors pertaining to speaking up and to compare hospital settings.

**Results:**

A total of 499 medical residents in the Netherlands participated in the study. Correlational analysis showed significant positive associations between each of support and control, and voice behaviour. The authors assumed that the associations between support and control, and voice behaviour would be partially mediated by engagement. This partial mediation model fitted the data best, but showed no association between support and voice. However, multi‐group analysis showed that for residents in hospital settings, support *is* associated with voice behaviour. For residents outside hospital settings, control is more important. Engagement mediated the effects of control and support outside hospital settings, but not within the hospital.

**Conclusions:**

This study shows that in order to enable medical residents to share their suggestions for improvement, it is beneficial to invest in *supportive supervision* and to increase their sense of *control*. Boosting medical residents’ support would be most effective in hospital settings, whereas in other health care organisations it would be more effective to focus on job control.

## Abstract 2

Job control and supervisor support are identified through this research as resources associated with residents “speaking up” and making suggestions for change, but the importance of each may depend on the setting in which residents work.

## Introduction

In health care organisations it is important that employees at all levels speak up and express their ideas on maintaining or increasing the quality of health care.^1^, ^2^ This is especially relevant for medical residents because they work at the frontline of patient care, in the context of which they experience and see both good and bad practice.^3^, ^4^, ^5^ Resident rotations allow them to visit many different departments, in which they can take a fresh look at work processes. As well as improving the quality of care, speaking up can lead to increased feelings of control over one's work, which can, in turn, lead to higher levels of well‐being.^6^ Further, the proactive sharing of suggestions for change is an important component of postgraduate medical education programmes as it is integrated into medical roles such as those of medical leadership and health advocacy.^7^, ^8^, ^9^ However, proactively speaking up about key points for improvement is not easy. It is considered to be ‘extra‐role behaviour’, which means that it goes beyond what is expected of employees and requires sufficient amounts of time and energy, both of which are scarce for residents.^3^, ^10^, ^11^ Moreover, employees who speak up can be viewed as tiring or strenuous, neither of which is favourable for residents.^12^ Thus, the benefits of speaking up are not self‐evident, especially not in the traditionally authoritarian health care context.^13^, ^14^, ^15^, ^16^ The purpose of this study is to identify and test which factors are associated with speaking up by medical residents.

### Speaking up by medical residents

In the social sciences, speaking up in order to exchange ideas, information or concerns that may benefit the organisation is often referred to as ‘voice behaviour’.^1^, ^12^, ^17^, ^18^, ^19^, ^20^ Three types of voice can be distinguished: the suggestion‐focused, the problem‐focused and the opinion‐focused voice.^1^, ^21^ Most research on speaking up by medical residents stems from the quality and safety literature, it describes speaking up with an expected *preventive* effect and refers to the problem‐focused voice. This voice makes expressions of concern about work practices, incidents or behaviours that can be harmful to the organisation, such as in speaking up about (un)professional behaviour, (hand) hygiene, ethical issues and risky or deficient actions on the part of medical staff.^14^, ^15^, ^18^, ^22^ In this study, we address a different type of speaking up, namely the use of the ‘suggestion‐focused voice’, which refers to the proactive communication of suggestions or ideas that might improve current work practices. Examples include the articulation of suggestions for changes in existing inefficient work routines, the pointing out of redundancies in administrative tasks and suggestions for the effective organisation of time, space and resources.^1^

Combining findings on speaking up from other professional fields with findings from research on the problem‐focused voice in medical residents, we argue that two basic considerations are important to medical residents who may wish to speak up and make suggestions for change. These concern: (i) whether it is safe to speak up (high support), and (ii) whether speaking up is likely to be effective (high control).^1^, ^12^, ^14^, ^15^, ^16^, ^17^, ^22^, ^23^ This is in line with well‐known behavioural models, such as the Job Demand–Control (–Support) (JDCS) Model, which argue that in highly demanding job contexts, high support and high perceived control over one's behaviour will lead to activation‐related outcomes such as motivation, learning and performance.^6^, ^24^, ^25^, ^26^, ^27^

#### Is it safe to speak up?

The social environment exerts a strong influence on a person's intentions and actions.^28^ A meta‐analysis on proactivity confirmed that social support is a major antecedent of proactive behaviour.^17^, ^29^ The receipt of support from peers or supervisors signals that an individual and his or her actions are accepted and valued.^13^, ^30^ We expect this relationship to be especially important in the medical context because of the close working relationships between residents and their supervisors. Thus, we expect supervisor support and speaking up to be positively associated (Hypothesis 1).

#### Is it effective to speak up?

Job control is also an important job characteristic in the literature on proactive behaviour and is associated with increased feelings of responsibility.^13^, ^17^ When employees feel control over situations, particularly if they feel they can influence work outcomes, their personal initiative may be increased.^13^ For residents, job control might refer to being able to influence current work routines. Therefore, we expect to find a positive association between job control and speaking up (Hypothesis 2).

#### The mediating role of work engagement

Work engagement is a ‘positive, fulfilling, work‐related state of mind that is characterised by vigour, dedication and absorption’.^31^, ^32^, ^33^, ^34^, ^35^ It is often studied as a form of well‐being. Similarly to speaking up, work engagement is contingent upon the presence of job resources such as control and support, but it also affects behaviour and performance at work.^32^ Work engagement may thus mediate the associations between job control, support and speaking up. Therefore, we expect that the effects of support and control on speaking up are at least partly mediated through engagement (Hypothesis 3).^31^

#### The influence of organisational context

Research on work behaviours in residents, such as burnout, engagement and workaholism, predominantly focuses on hospital residents. Little is known about residents who work in the settings of other health care organisations, such as in the public health sector.^36^, ^37^, ^38^ We hypothesise that there are cultural and contextual differences related to safety and support between work contexts within and outside hospital settings. Consequently, we explore whether the studied relationships are different for residents who work in hospital settings compared with residents outside hospital settings.

**Figure 1:**
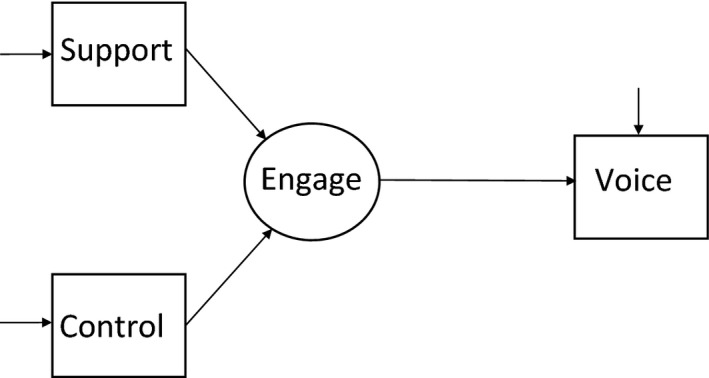
Full mediation model (M_1_) for the associations between supervisor support, job control, work engagement and voice behaviour

In summary, this study aims to explore whether two main aspects of medical residents’ work context, *job control* and *supervisor support*, are associated with speaking up by medical residents. Moreover, we examine whether these associations are mediated by residents’ work engagement. Figure 1 represents the hypothesised research model (M_1_). This paper will test the hypothesised model using structural equation modelling (SEM). In SEM, a researcher specifies a model based on existing theory and then tests this model by simultaneously analysing the *entire* system of relationships amongst the study variables. When doing so, the researcher analyses the extent to which the model is consistent with the data (i.e. its goodness‐of‐fit, as expressed in fit indices).^39^

## Methods

### Design and participants

We tested the hypothesised model using a cross‐sectional survey design. The survey was distributed amongst residents in the Netherlands during March–May 2018. Participants were approached by means of newsletters, direct e‐mails, links in learning environments and social media. Of the 580 respondents, 81 cases were deleted for reasons of (partly) missing data. Of the remaining 499 participants, 70% were female. The mean ± standard deviation (SD) age of the sample was 33 ± 6.1 years. For the multi‐group analysis, respondents were assigned to an in‐ or out‐of‐hospital group, based on their current positions (Table 1). Of the respondents, 299 currently worked in hospital settings and 200 worked in other health care organisations such as mental health care centres, public health centres and occupational health agencies.

**Table 1:** Characteristics of residents (*n* = 499) working in or outside hospital settings

	Residents working in hospital settings, *n*	Residents working outside hospital settings, *n*
All residents	299	200
Specialty type*		
Cluster 1: general physicians, elderly care physicians, physicians for patients with learning disabilities	2	4
Cluster 2: hospital specialties	282	53
Cluster 3: public health physicians	–	142
Missing data on specialty training programme	15	1
Current organisation		
General affiliated teaching hospital	124	
Academic medical centre	174	
Other in‐hospital setting	1	
Mental health centre		50
Public health centre		70
Nursing home		4
Employee service agency		18
Occupational health agency		16
Centre for youth and development		7
Rehabilitation centre		3
Other (e.g. politics, insurance company, commercial business)		32

* In the Netherlands, residents are divided into three clusters. Cluster 1 represents residency training programmes for general physicians, elderly care physicians and physicians for patients with intellectual disabilities. Cluster 2 covers residency programmes for hospital physicians such as surgeons, neurologists, paediatricians, radiologists etc. Cluster 3 represents residency training programmes for public health physicians.

As we did not know how many potential participants had been reached by our efforts, it was impossible to compute a response rate. However, our sample included 5% of the total population of residents in the Netherlands. To account for response bias, we compared our participant group with the general population of residents in the Netherlands for age, gender and organisation type. Of the respondents, 60% worked in hospital settings and 40% worked outside the hospital (e.g. in community health centres or mental health care facilities). This equates to the distribution of residents across in‐ and out‐of‐hospital settings in the Netherlands (60% and 40%, respectively).^40^ Moreover, we checked whether the means and SDs of work engagement in our sample were comparable with those of a large study (*n *=* *2114; response rate: 41%) on work engagement in Dutch medical residents^38^ and found a strong degree of similarity. Thus, we believe that our sample is largely representative for the topic of work engagement in the total population of residents in the Netherlands.

This study falls outside the scope of the Dutch Medical Research Involving Human Subjects Act (WMO) and therefore ethical approval was not formally requested. However, we did protect our participants. The residents were informed that participation was both voluntary and anonymous and that it was possible to withdraw from the survey at any time. All study materials were anonymised and saved by one researcher (JV) on a protected server.

### Measures

#### Speaking up

We operationalised speaking up as suggestion‐focused voice*,* which was measured with six items taken from the work of van Dyne and LePine.^20^ The original items were translated into Dutch by a native translator using back‐and‐forth translation. Because our questionnaire was based on self‐reports, the words ‘this employee’ were replaced by ‘I’, such as in the example item: ‘I speak up in this group with ideas for new projects or changes in procedures.’ Responses to items were given on a scale of 1 (never) to 7 (always). Appendix S1 provides an English‐language version of the complete survey.

#### Control

We measured c*ontrol* using the 10‐item ‘Influence at work’ scale of the Copenhagen Psychosocial Questionnaire.^41^ An example item is: ‘Do you have a large degree of influence concerning your work?’

#### Supervisor support

We measured *supervisor support* using the eight items of the Supportive Supervision Scale.^42^ An example item is: ‘My supervisor encourages employees to speak up when they disagree with a decision.’

#### Work engagement

We measured *work engagement* as a three‐factor model using the nine items of the Utrecht Work Engagement Scale.^33^, ^43^ Three items tapped vigour (e.g. ‘At my work, I feel bursting with energy’), three items tapped dedication (e.g. ‘I am enthusiastic about my job’) and three tapped absorption (e.g. ‘I feel happy when I am working intensely’). Work engagement has been extensively studied and previous confirmatory factor analyses showed that a three‐factor model was superior to a one‐factor model.^32^

#### Background variables

Background variables included age, gender, specialty training programme, year of training, work experience and organisation.

### Statistical analyses

#### Preliminary analyses

We checked the data for normality. Reliability estimates showed good internal consistency for all scales (Table 2), except ‘supportive supervision’. We deleted one item as a result of a low factor loading and a negative association with ‘voice’ (i.e. ‘My supervisor refuses to explain his or her actions’), which resulted in a scale of seven items with good internal consistency. We performed a Harman single‐factor test to account for common method variance.^44^

**Table 2:** Means, standard deviations (SDs), correlations and reliabilities (Cronbach's alpha, on the diagonal) of the study variables in data for 499 residents in the Netherlands, 2018

	Mean	SD	1	2	3	4	5	6	7	8
1 Voice	4.61	1.02	0.91							
2 Support	4.33	1.11	0.27†	0.90						
3 Control	3.84	0.92	0.37†	0.48†	0.89					
4 Absorption	3.92	1.00	0.18†	0.32†	0.26†	–				
5 Dedication	4.56	0.87	0.17†	0.40†	0.30†	0.70†	–			
6 Vigour	4.05	0.92	0.30†	0.38†	0.37†	0.63†	0.70†	–		
*Demographic variables*										
7 Age	33	6.1	0.10*	−0.09*	0.10*	−0.08	−0.10*	0.02	−	
8 Gender, female	0.79	0.41	0.00	0.01	−0.01	−0.00	−0.02	0.01	−0.02	−

* p ≤ 0.05.

† p ≤ 0.01.

#### Main analyses

We examined the research model using SEM in MPlus Version 8.1 (Muthén & Muthén, Los Angeles, CA, USA). Control, support and voice were treated as observed variables (using the means of corresponding scales) and work engagement was treated as a latent variable with dedication, vigour and absorption as its three indicators. Missing data were handled using full information maximum likelihood methods. Model fit was assessed using the chi‐squared statistic, the Tucker–Lewis index (TLI) (> 0.90 indicates acceptable fit), the root mean square error of approximation (RMSEA) (< 0.08 indicates mediocre fit, < 0.05 indicates good fit) and the Akaike information criterion (AIC).^39^ For the mediation analysis, we applied a bootstrapping procedure. We calculated the total indirect effects of support and control on voice through work engagement to examine possible mediation effects.^45^ For the multi‐group analysis, we compared the model fit of a constrained model (all parameters were constrained to be equal across both groups) with the fit of an unconstrained model (with parameters differing across groups). To assess the strength of our results compared with previous findings amongst other professional groups, we used the pooled data from a meta‐analysis on voice and compared the model fit using their effect sizes compared with ours.^17^

## Results

### Correlational analysis

Table 2 presents the means, correlations and reliabilities of the study variables. There were positive associations between support (*r *=* *0.27, p* *<* *0.01) and control (*r *=* *0.37, p* *<* *0.01) with voice, confirming Hypotheses 1 and 2. The Harman single‐factor test did not provide strong indications for common method variance, as the explained variance by a single factor was < 50% (33.7%).

**Table 3:** Means and correlations of the study variables compared between hospital residents (*n *= 299, below the diagonal line) and residents working outside hospital settings (*n *=* *200*,* above the diagonal line) in 499 residents in the Netherlands in 2018

	Mean in hospital	Mean outside hospital	1	2	3	4	5	6	7	8
1 Voice	4.4‡	4.9‡	1	0.13	0.29†	0.19†	0.21†	0.31†	0.04	0.03
2 Support	4.3	4.4	0.39†	1	0.51†	0.29†	0.37†	0.27†	−0.12	0.03
3 Control	3.5‡	4.3‡	0.34†	0.51†	1	0.30†	0.36†	0.39†	−0.10	−0.12
4 Absorption	3.9	3.9	0.19†	0.36†	0.29†	1	0.65†	0.55†	−0.08	0.06
5 Dedication	4.6	4.5	0.17†	0.44†	0.36†	0.72†	1	0.65†	−0.08	0.02
6 Vigour	4.0	4.1	0.29†	0.47†	0.40†	0.68†	0.74†	1	0.07	0.02
*Demographic variables*										
7 Age	30.8‡	36.2‡	−0.01	−0.13*	−0.10	−0.09	−0.03*	−0.00	1	−0.10
8 Gender, female	0.77	0.83	−0.04	−0.01	0.00	−0.03	−0.03	−0.00	−0.02	1

* p ≤ 0.05.

† p ≤ 0.01.

‡ Significant difference between groups.

### Structural equation analysis

The full mediation model (M_1_) fitted the data only marginally (χ^2^[degrees of freedom, d.f.* = *8] = 74.318; RMSEA = 0.129, 90% confidence interval [CI] 0.103–0.156; TLI = 0.837; AIC = 7259.280). The partial mediation model (M_2_) fitted the data considerably better (χ^2^[d.f.* *=* *6] = 27.211; RMSEA = 0.084, 90% CI 0.054–0.117; TLI = 0.946; AIC = 7216.173). The difference in χ^2^‐values between the competing models was significant (Δχ^2^[d.f.* = *2] = 47.107; p* *<* *0.01), which indicates that the partial mediation model is preferred to the full mediation model. Appendix S2 shows the path coefficients of the partial mediation model. We found a significant association between control and voice (0.29, p* *<* *0.01), but not between support and voice (0.08, p* *>* *0.05). Our results showed that work engagement does not mediate the effects of job control and supervisor support on voice behaviour within the full sample and reject Hypothesis 3 (0.11, p* *>* *0.05).

To assess the relative importance of control and support for residents compared with other employees, we compared our path coefficients with the pooled effect sizes in previous research (0.37 versus 0.20 for control and 0.27 versus 0.15 for support).^17^ We found that for control, our study reports significantly stronger associations compared with previous studies (Δχ^2^ = 4.761, Δd.f. = 1, p* *=* *0.03).

### Multi‐group comparison

**Figure 2:**
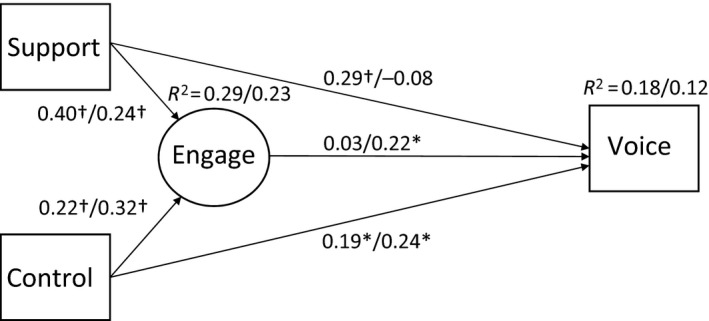
Structural paths from the multi‐group analysis of the partial mediation model (M_2_). Coefficients represent standardised estimates for hospital residents (*n* = 299)/residents working outside hospital settings (*n* = 200). Total *n* = 499 residents, the Netherlands, 2018. *, p* *<* *0.05; †, p* *<* *0.01

Table 3 shows the means and correlations of the study variables compared between residents working in‐ or outside hospital settings. Multi‐group SEM analysis showed that the model results differ between the resident groups (Δχ^2^ = 28.927 [d.f.* *=* *8], p* *<* *0.01). The path coefficient between voice and support was significant for hospital residents (0.29, p* *<* *0.01), but not for residents who work outside hospital settings (−0.08, p* = *0.32). Moreover, the path coefficient between engagement and voice was significant for residents who work outside hospital settings (0.22, p* *<* *0.05) but not for hospital residents (0.03, p* *=* *0.70) (Fig. 2). Mediation analysis showed that work engagement partly mediates the effect between control, support and voice for residents outside hospital settings (0.08, standard error [SE] = 0.04 [95% CI 0.02–0.19] and 0.04, SE = 0.02 [95% CI 0.01–0.10], respectively).

## Discussion

This study showed that both job control and supervisor support are job resources that are associated with speaking up by medical residents. However, their associations differ across settings. *Support* was an important resource for speaking up for hospital residents, whereas work engagement had no significant mediating effect. For residents outside hospital settings, *control* was an important resource for speaking up. In this group, work engagement positively related to speaking up and partially mediated the effect of control. Although we tested only for associations, previous studies on job resources and active work behaviours such as speaking up support the proposed direction of the effect as depicted in the research model.^6^, ^26^, ^32^, ^46^, ^47^, ^48^, ^49^

We found that the relationship between control and voice is stronger in our study than in other work settings. Thus, control is a relatively important resource for residents.^17^ A possible explanation for the absent associations between work engagement and speaking up by medical residents in hospital settings is that contextual factors might inhibit residents from voicing their opinions, which may overwhelm their levels of engagement. One such contextual factor could be the frequent change of work environment that results from the rotational character of in‐hospital specialty training programmes. Residents spend only a few months in a specific department before switching to the next. This may negatively influence their motivation to speak up as previous research shows that speaking up is positively related to longer organisational tenure and experience.^1^, ^50^ It is possible that hospital residents feel they lack the time or credibility to make effective suggestions for change, which also relates to a lower sense of control.

Our results show that supervisor support is important for medical residents, but does not influence speaking up by residents outside hospital settings. Hospital residents generally work closely with their supervisors in a ‘master–apprentice’‐like context. In hospitals, physicians are socialised through what is referred to as the ‘hidden curriculum’ in an informal learning process in which novices learn how to behave according to professional and occupational standards.^51^, ^52^ Hierarchy is an important element of this curriculum. This may explain why the support of supervisors is especially important for hospital residents when trying to speak up. Outside hospital settings, medical residents usually spend more time in the same department or organisation, and thus build stronger networks and are less dependent on their supervisors. This may explain why support and speaking up are not significantly related for ut‐of‐hospital residents as the influence of a direct supervisor may be less important in a stronger network. In our sample, female residents were slightly over‐represented (79% in our sample versus 71% in the Dutch population) and our respondents were, on average, slightly younger (33 years versus 34 years) than the total population of residents in the Netherlands. Note that the literature provides no conclusive evidence on the influence of gender on speaking up and we did not find any significant correlations between gender and our variables of interest.^1^

### Limitations

One limitation of the current study is its low response rate, which may have resulted in non‐response bias. We calculated the response rate based on *all* residents in the Netherlands because we used social media as part of our distribution strategy. However, it is unlikely that we reached all residents via these communication channels, meaning that our actual response rate is higher.

Further, the cross‐sectional design means that our results must be interpreted as associations rather than causal relationships. Moreover, as is common in behavioural research, we were able to explain only a relatively small part of the variance of the study concepts. In line with the literature on proactive behaviour at work, we focused on two main factors: job control and supervisor support. However, it is likely that other variables that were not included in our study also influence speaking up by residents.

### Implications

Our results provide starting points for medical education programmes to enable residents to speak up and make suggestions for change, using specific organisational and occupational interventions that are targeted towards increasing residents’ sense of control and support. For example, it may be worthwhile to train supervisors in supporting residents in stepping forward with suggestions for change, thereby creating a positive learning climate. When speaking up becomes part of local culture, the threshold at which residents will step forward with their suggestions is lowered. We do not believe (nor do we think it would be beneficial to organisations) that employees should be able to speak up about each and every issue they come across. We do believe that there is a minimal level at which residents should be able to speak up. Moreover, enhancing residents’ sense of control such as by involving them in staff meetings and think‐tanks or by simply asking for their opinions could stimulate them to speak up. This is different from most current postgraduate medical education strategies, which are more focused on individual (competency) training.^53^ When local training programmes acknowledge that speaking up can be difficult for residents who are junior employees, this acknowledgement represents an important first step in creating a culture of speaking up and sharing novel ideas, suggestions and experiences.

Theoretically, this study further strengthens the evidence that control and support are positively associated with speaking up by (medical) employees. This association differs between hospital residents and residents who work outside hospital settings. Moreover, we demonstrated that work engagement is associated with job resources, which is in line with the findings of previous research.^1^, ^17^, ^20^, ^54^

## Conclusions

This study showed that perceived control and support are associated with speaking up and sharing suggestions for change by medical residents. This emphasises the suggestion that residents do not act in a vacuum; rather, they are embedded in their professional and organisational contexts. This embeddedness calls for attention to contextual factors such as job control and supervisor support that positively influence residents to speak up and share their ideas for change.

## Contributors

JJV, TWT and MFvdS contributed to the theoretical and statistical design, the analysis and interpretation of the data and the preparation of the manuscript. ELJvR, MMES and MN contributed to the design of the study, the interpretation of results and the critical review of the manuscript. All authors (JJV, TWT, ELJvR, MMES, MN and MFvdS) approved the final manuscript for submission.

## Funding

none.

## Conflicts of interest

none.

## Ethical approval

not required.

## Supporting information


**Appendix S1.** Survey guide.

**Appendix S2.** Path coefficients of the partial mediation model.
