# Supplementary material for: Speaking up, support, control and work engagement of medical residents. A structural equation modelling analysis
Source: Med Educ. 2019 Sep 30;53(11):1111–20. doi: 10.1111/medu.13951 (PMC6856833; doi:10.1111/medu.13951)
Supplement: Supplementary file 1 — Appendix S1. Survey guide. [file MEDU-53-1111-s001.docx]

**APPENDIX 1: SURVEY GUIDE**

**Includes:**

1. Utrecht work engagement scale – 9 items
   *Source: Wilmar Schaufeli & Arnold Bakker – Utrechtse Bevlogenheid Schaal, Voorlopige handleiding (versie oktober 2003)*
2. Big Five Inventory – 10 items^*^
   *Sources: Denissen, J.J.A., Geenen, R., & van Aken, M. A. G., Gosling, S. D., & Potter, J. (2008). Development and validation of a Dutch translation of the Big Five Inventory (BFI). Journal of Personality Assessment, 90, 152-157.*

*Rammstedt, B. & John, O. P. (2007). Measuring personality in one minute or less: A 10-item short version of the Big Five Inventory in English and German. Journal of Research in Personality, 41, 203-212.*

1. Voice – 6 items
   *Source: Helping and Voice Extra-Role Behaviors: Evidence of Construct and Predictive Validity. Linn Van Dyne and Jeffrey A. LePine The Academy of Management JournalVol. 41, No. 1 (Feb., 1998), pp. 108-119)*
2. Job Control – 10 items
   *Kristensen, T.F., & Borg, V. (2003). Copenhagen Psychosocial Questionnaire (COPSOQ). Copenhagen: AMI.*
3. Supportive supervision – 8 items
   *Oldham, G.R., & Cummings, A (1996) employee creativity: personal and contextual factors at work. Academy of Mangement Journal, 39(3), 607-634.*

*The Big Five personality questionnaire was added to the survey, but not used in the analyses

**Work engagement (UWES-9)**

| 1. | At my work, I feel bursting with energy. | 1 | 2 | 3 | 4 | 5 | 6 | 7 |
| --- | --- | --- | --- | --- | --- | --- | --- | --- |
| 2. | At my job, I feel strong and vigorous. | 1 | 2 | 3 | 4 | 5 | 6 | 7 |
| 3. | I am enthusiastic about my job. | 1 | 2 | 3 | 4 | 5 | 6 | 7 |
| 4. | My job inspires me. | 1 | 2 | 3 | 4 | 5 | 6 | 7 |
| 5. | When I get up in the morning, I feel like going to work | 1 | 2 | 3 | 4 | 5 | 6 | 7 |
| 6. | I feel happy when I am working intensely. | 1 | 2 | 3 | 4 | 5 | 6 | 7 |
| 7. | I am proud of the work that I do. | 1 | 2 | 3 | 4 | 5 | 6 | 7 |
| 8. | I am immersed in my work. | 1 | 2 | 3 | 4 | 5 | 6 | 7 |
| 9. | I get carried away when I am working | 1 | 2 | 3 | 4 | 5 | 6 | 7 |

1 = never, 7 = always

### Big Five Inventory

### I see myself as someone who..

| 1. | ... Is reserved * | 1 | 2 | 3 | 4 | 5 |
| --- | --- | --- | --- | --- | --- | --- |
| 2. | ... Is generally trusting. | 1 | 2 | 3 | 4 | 5 |
| 3. | ... Tends to be lazy. * | 1 | 2 | 3 | 4 | 5 |
| 4. | ... Is relaxed, handles stress well.* | 1 | 2 | 3 | 4 | 5 |
| 5. | ... Has few artistic interests.* | 1 | 2 | 3 | 4 | 5 |
| 6. | ... Is outgoing, sociable. | 1 | 2 | 3 | 4 | 5 |
| 7. | ... Tends to find fault with others * | 1 | 2 | 3 | 4 | 5 |
| 8. | ... Does a thorough job. | 1 | 2 | 3 | 4 | 5 |
| 9. | ... Gets nervous easily | 1 | 2 | 3 | 4 | 5 |
| 10 | … Has an active imagination | 1 | 2 | 3 | 4 | 5 |

### 1 = disagree, 5 = totally agree

**Voice behavior**

| 1. | I develop and make recommendations concerning issues that affect this work group. | 1 | 2 | 3 | 4 | 5 | 6 | 7 |
| --- | --- | --- | --- | --- | --- | --- | --- | --- |
| 2. | I speak up and encourage others in this group to get involved in issues that affect the group | 1 | 2 | 3 | 4 | 5 | 6 | 7 |
| 3. | I communicate my opinions about work issues to others in this group even if my opinion is different and others in the group disagree with me | 1 | 2 | 3 | 4 | 5 | 6 | 7 |
| 4. | I keep well informed about issues where my opinion might be useful to this work group | 1 | 2 | 3 | 4 | 5 | 6 | 7 |
| 5. | I get involved in issues that affect the quality of work life here in this group. | 1 | 2 | 3 | 4 | 5 | 6 | 7 |
| 6. | I speak up in this group with ideas for new projects or changes in procedures. | 1 | 2 | 3 | 4 | 5 | 6 | 7 |

1 = never, 7 = always

**Job control** (influence at work)

| 1. | Do others decide on (parts of) your work? | 1 | 2 | 3 | 4 | 5 | 6 | 7 |
| --- | --- | --- | --- | --- | --- | --- | --- | --- |
| 2. | Do you have a large degree of influence concerning your work? | 1 | 2 | 3 | 4 | 5 | 6 | 7 |
| 3. | Can you influence how quickly you work? | 1 | 2 | 3 | 4 | 5 | 6 | 7 |
| 4. | Do you have a say in choosing who you work with? | 1 | 2 | 3 | 4 | 5 | 6 | 7 |
| 5. | Can you influence the amount of work assigned to you? | 1 | 2 | 3 | 4 | 5 | 6 | 7 |
| 6. | Can you organize your own work? | 1 | 2 | 3 | 4 | 5 | 6 | 7 |
| 7. | Do you have any influence on how you do your work? | 1 | 2 | 3 | 4 | 5 | 6 | 7 |
| 8. | Do you have any influence on what you do at work? | 1 | 2 | 3 | 4 | 5 | 6 | 7 |
| 9. | Can you influence your work environment? | 1 | 2 | 3 | 4 | 5 | 6 | 7 |
| 10. | Do you have any influence on the quality of the work? | 1 | 2 | 3 | 4 | 5 | 6 | 7 |

1 = never, 7 = always

**Supportive supervision**

| 1. | In general, my supervisors help me solve work-related problems, | 1 | 2 | 3 | 4 | 5 | 6 | 7 |
| --- | --- | --- | --- | --- | --- | --- | --- | --- |
| 2. | In general, my supervisors encourage me to develop new skills. | 1 | 2 | 3 | 4 | 5 | 6 | 7 |
| 3. | In general, my supervisors keep informed about how employees think and feel about things | 1 | 2 | 3 | 4 | 5 | 6 | 7 |
| 4. | In general, my supervisors encourage employees to participate in important decisions | 1 | 2 | 3 | 4 | 5 | 6 | 7 |
| 5. | In general, my supervisors praise good work. | 1 | 2 | 3 | 4 | 5 | 6 | 7 |
| 6. | In general, my supervisors encourage employees to speak up when they disagree with a decision | 1 | 2 | 3 | 4 | 5 | 6 | 7 |
| 7. | In general, my supervisors refuse to explain their actions* | 1 | 2 | 3 | 4 | 5 | 6 | 7 |
| 8. | In general, my supervisors reward me for good performance. | 1 | 2 | 3 | 4 | 5 | 6 | 7 |

1 = never, 7 = always
