# Supplementary figures and images for: Speaking up, support, control and work engagement of medical residents. A structural equation modelling analysis
Source: Med Educ. 2019 Sep 30;53(11):1111–20. doi: 10.1111/medu.13951 (PMC6856833; doi:10.1111/medu.13951)

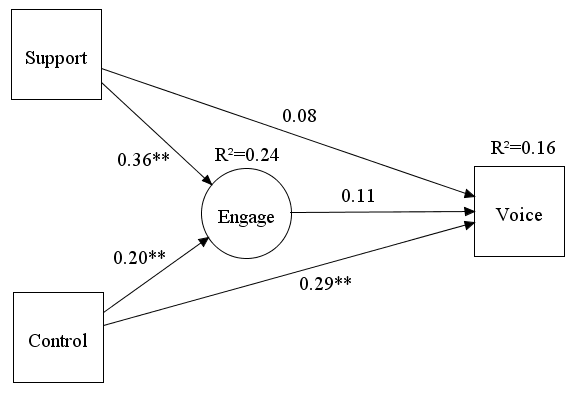

Supplement: Supplementary file 2 — Appendix S2. Path coefficients of the partial mediation model. [file MEDU-53-1111-s002.png]
